# 

**Published:** 1967-04

**Authors:** 


### *Contents*

| | | Page |
|---|---|---|
| CAVE DIVING HAZARDS ........................ | *Oliver C. Lloyd* | 23 |
| PICTORIAL ART IN MEDICINE ........... | *G. M. Fitzgibbon* | 35 |
| HAEMOLYTIC DISEASE OF THE NEWBORN WITH PULMONARY ATELECTASIS: A Clinico-Pathological Conference | | 46 |
| ON THE CHANGING FACE OF RHEUMATISM | *Professor C. B. Perry* | 54 |
| LONG TERM PROGNOSIS IN CLOSED HEAD INJURY | *C. Adey* | 58 |

